# Molecular diversity and frequency of the diarrheagenic enteric protozoan *Giardia duodenalis* and *Cryptosporidium* spp. in a hospital setting in Northern Spain

**DOI:** 10.1371/journal.pone.0178575

**Published:** 2017-06-15

**Authors:** José Manuel Azcona-Gutiérrez, Aida de Lucio, Marta Hernández-de-Mingo, Concepción García-García, Luis Miguel Soria-Blanco, Lucía Morales, María Aguilera, Isabel Fuentes, David Carmena

**Affiliations:** 1 Department of Biomedical Diagnostics, Hospital San Pedro, Logroño, La Rioja, Spain; 2 Parasitology Service, National Centre for Microbiology, Majadahonda, Madrid, Spain; 3 Department of Infectious Diseases, Hospital San Pedro, Logroño, La Rioja, Spain; Food and Drug Administration, UNITED STATES

## Abstract

**Background:**

Human giardiosis and cryptosporidiosis are caused by the enteric protozoan parasites *Giardia duodenalis* and *Cryptosporidium* spp. Both pathogens are major contributors to the global burden of diarrhoeal disease, affecting primarily children and immunodebilitated individuals in resource-poor settings. Giardiosis and cryptosporidiosis also represent an important, often underestimate, public health threat in developed countries. In Spain only limited information is currently available on the epidemiology of these infections. Molecular data on the diversity, frequency, geographical distribution, and seasonality of *G*. *duodenalis* assemblages/sub-assemblages and *Cryptosporidium* species/sub-genotypes are particularly scarce.

**Methods:**

A longitudinal molecular epidemiological survey was conducted between July 2015 to September 2016 in patients referred to or attended at the Hospital San Pedro (La Rioja, Northern Spain) that tested positive for *G*. *duodenalis* (N = 106) or *Cryptosporidium* spp. (N = 103) by direct microscopy and/or a rapid lateral flow immunochromatographic assay. *G*. *duodenalis* infections were subsequently confirmed by real-time PCR and positive isolates assessed by multi-locus sequence genotyping of the glutamate dehydrogenase and β-giardin genes of the parasite. *Cryptosporidium* species and sub-genotypes were investigated at the 60 kDa glycoprotein or the small subunit ribosomal RNA genes of the parasite. Sociodemographic and clinical parameters of infected patients were also gathered and analysed.

**Principal findings:**

Out of 90 *G*. *duodenalis*-positive isolates by real-time PCR a total of 16 isolates were successfully typed. AII (44%, 7/16) was the most prevalent sub-assemblage found, followed by BIV (31%, 5/16) and BIII (19%, 3/16). A discordant genotype result AII/AIII was identified in an additional (6%, 1/16) isolate. No mixed infections A+B were detected. Similarly, a total of 81 *Cryptosporidium* spp. isolates were successfully typed, revealing the presence of *C*. *hominis* (81%, 66/81) and *C*. *parvum* (19%, 15/81). Obtained *GP60* sequences were assigned to sub-type families Ib (73%, 59/81) within *C*. *hominis*, and IIa (7%, 6/81) and IId (2%, 2/81) within *C*. *parvum*. A marked inter-annual variation in *Cryptosporidium* cases was observed.

**Conclusions:**

Human giardiasis and cryptosporidiosis are commonly identified in patients seeking medical care in Northern Spain and represent a more important health concern than initially thought. Assemblage A within *G*. *duodenalis* and sub-genotype IbA10G2 within *C*. *hominis* were the genetic variants of these parasite species more frequently found circulating in the population under study. Molecular data presented here seem to suggest that *G*. *duodenalis* and *Cryptosporidium* infections arise through anthroponotic rather than zoonotic transmission in this Spanish region.

## Introduction

The enteric protozoan *Giardia duodenalis* and *Cryptosporidium* spp. are the etiological agents of human giardiosis and cryptosporidiosis. Both parasite species, particularly the later, are considered emerging opportunistic pathogens responsible for a significant proportion of diarrhoeal morbidity globally [1,2]. Giardiosis and cryptosporidiosis are highly prevalent and widespread diseases that primarily affect young children with vulnerable socio-economic conditions in developing countries. In these unfavourable settings early childhood giardiosis/cryptosporidiosis has been linked with malnutrition, growth faltering, and cognitive deficits [3,4]. Additionally, *Cryptosporidium* spp. has been identified as a leading pathogen associated with death in toddlers [5].

Both giardiosis and cryptosporidiosis are transmitted by the faecal-oral route, either directly through person-to-person (anthroponotic) or animal-to-person (zoonotic) contact, or indirectly through contaminated drinking/recreational water or food. Indeed, waterborne [6] and foodborne [7] outbreaks of giardiosis/cryptosporidiosis have been increasingly documented in recent years, mainly in developed countries. *G*. *duodenalis* consists of eight (A to H) genetically distinct genotypes (assemblages) with variable host ranges and specificities. Assemblages A and B are the only two assemblages known to consistently infect humans, among other mammal species, and are therefore considered to be zoonotic [8]. Similarly, at least 30 valid species and over 70 genotypes of uncertain taxonomic status have been identified within the genus *Cryptosporidium* to date [9]. Over 90% of all reported human infections are caused by the anthroponotic *C*. *hominis* and the zoonotic *C*. *parvum* [9]. Other *Cryptosporidium* species including *C*. *meleagridis*, *C*. *canis*, *C*. *felis*, *C*. *ubiquitum*, and *C*. *cuniculus* are less frequently or only sporadically detected in humans [10–12].

In developed countries the prevalence of *G*. *duodenalis* has been reported in the range of 2%‒7%, whereas it is estimated that *Cryptosporidium* infections account for up to 9% of diarrhoeal episodes in children [13]. Giardiosis and cryptosporidiosis are subjected to mandatory surveillance and notification in most European Union and European Economic Area countries [14]. However, both diseases are severely under-diagnosed and under-reported due a combination of factors including health care seeking behaviour by patients, poor awareness of general practitioners in primary health centres, low request rate for specific testing, variable provision of diagnostic tests, and lack of harmonized monitoring programs [15]. In Spain, reported human infection rates by *G*. *duodenalis* and *Cryptosporidium* spp. are well in the range of those documented in other developed countries [16,17]. The diversity and population structure of these pathogens have been investigated in a limited number of molecular epidemiological surveys. These studies revealed that assemblage B, particularly sub-assemblage BIV, was the most prevalent *G*. *duodenalis* assemblage circulating in Spanish human populations [18–22], whereas a general predominance of *C*. *hominis* over *C*. *parvum* [23–27] together with the occasional presence of *C*. *meleagridis* [24,26–28], *C*. *felis* [23,24,28,29], *C*. *ubiquitum* [29] or *C*. *cuniculus* [12] has been documented within the genus *Cryptosporidium*.

We present here novel data on the presence, distribution, frequency, and molecular diversity of *G*. *duodenalis* and *Cryptosporidium* spp. during a 15-month period in the Autonomous Region of La Rioja, a Spanish geographical area where the epidemiology of these pathogens have not been previously investigated.

## Materials and methods

### Ethical statement

Patient informed consents were not required since the stool specimens used in this survey were exclusively intended for routine clinical diagnostic procedures. The identified information of the patients, including socio-demographic or clinical data, were conveniently anonymized to preserve the identity of the individuals involved. The design of the project, the method assurance of patient confidentiality, and the waiving of informed consent documentation have been approved by the Research Ethics Committee of the Carlos III Health Institute (reference number: CEI PI 34_2014).

### Area, population, and study design

The autonomous region of La Rioja (Norther Spain) has an area of 5,045 km^2^ and a total population exceeding 319,000, of which approximately half are living in the capital city, Logroño. The Hospital San Pedro (HSP) is the major public, general hospital in the region with 630 inpatient beds available, serving 16 out of the 20 catchment districts (health areas) in which La Rioja is administratively divided.

**Fig 1:**
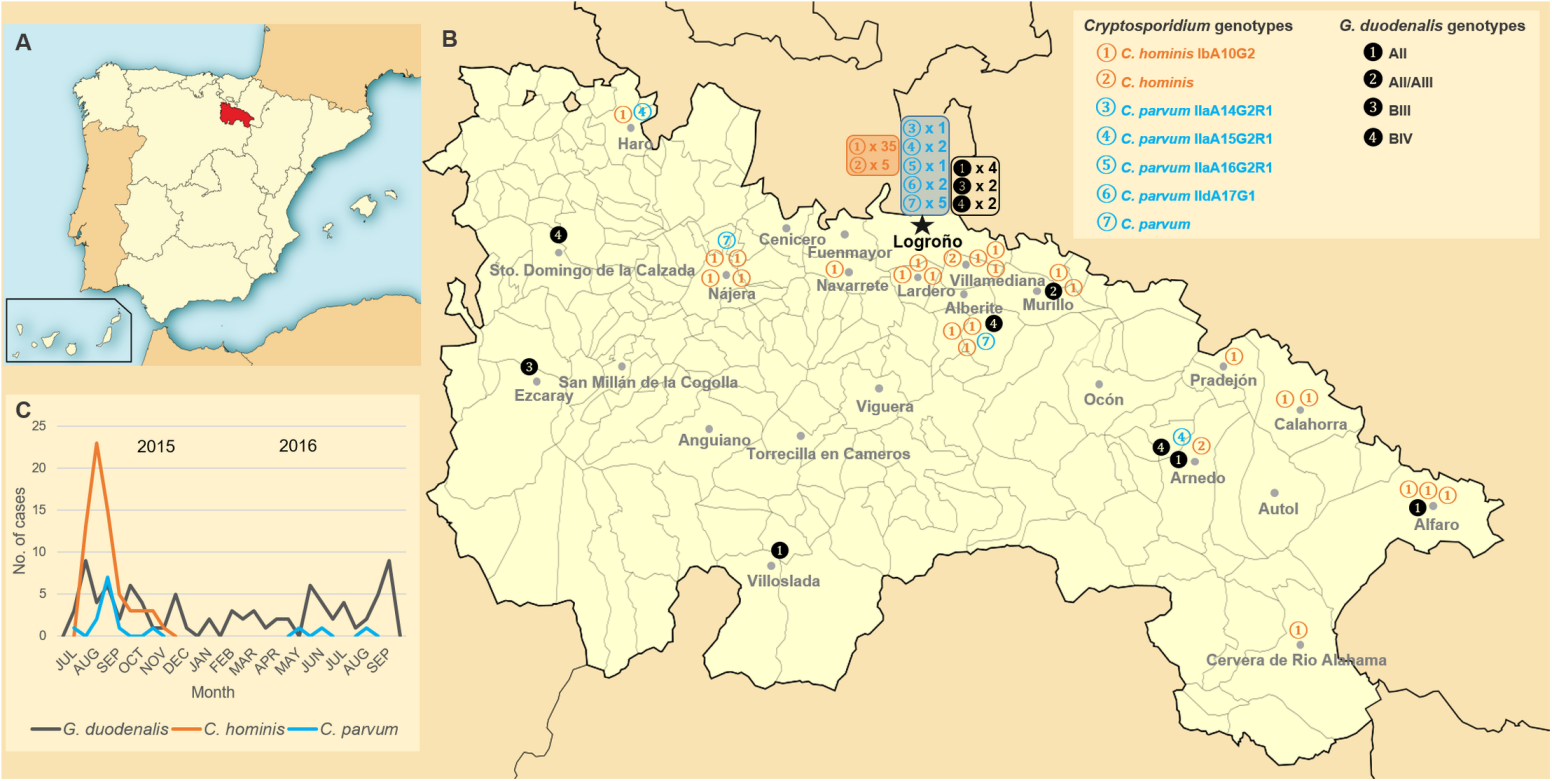
Temporal and geographical distribution of *G*. *duodenalis* and *Cryptosporidium* spp. infections in La Rioja, Northern Spain (2015–2016). Panel A: Map of Spain indicating the exact location of the Autonomous region of La Rioja. Panel B: Distribution of *G*. *duodenalis* and *Cryptosporidium* cases according to the primary health care centre of origin and the assemblage/genotype of the parasite species considered. A genotype identification key is provided in the upper right corner of the panel. Panel C: Temporal distribution of *G*. *duodenalis*, *C*. *hominis* and *C*. *parvum* infections through the period of study. An identification key for species and sub-genotypes is provided at the bottom of the panel. Reprinted from Wikimedia Commons, the free media repository.

**Fig 2:**
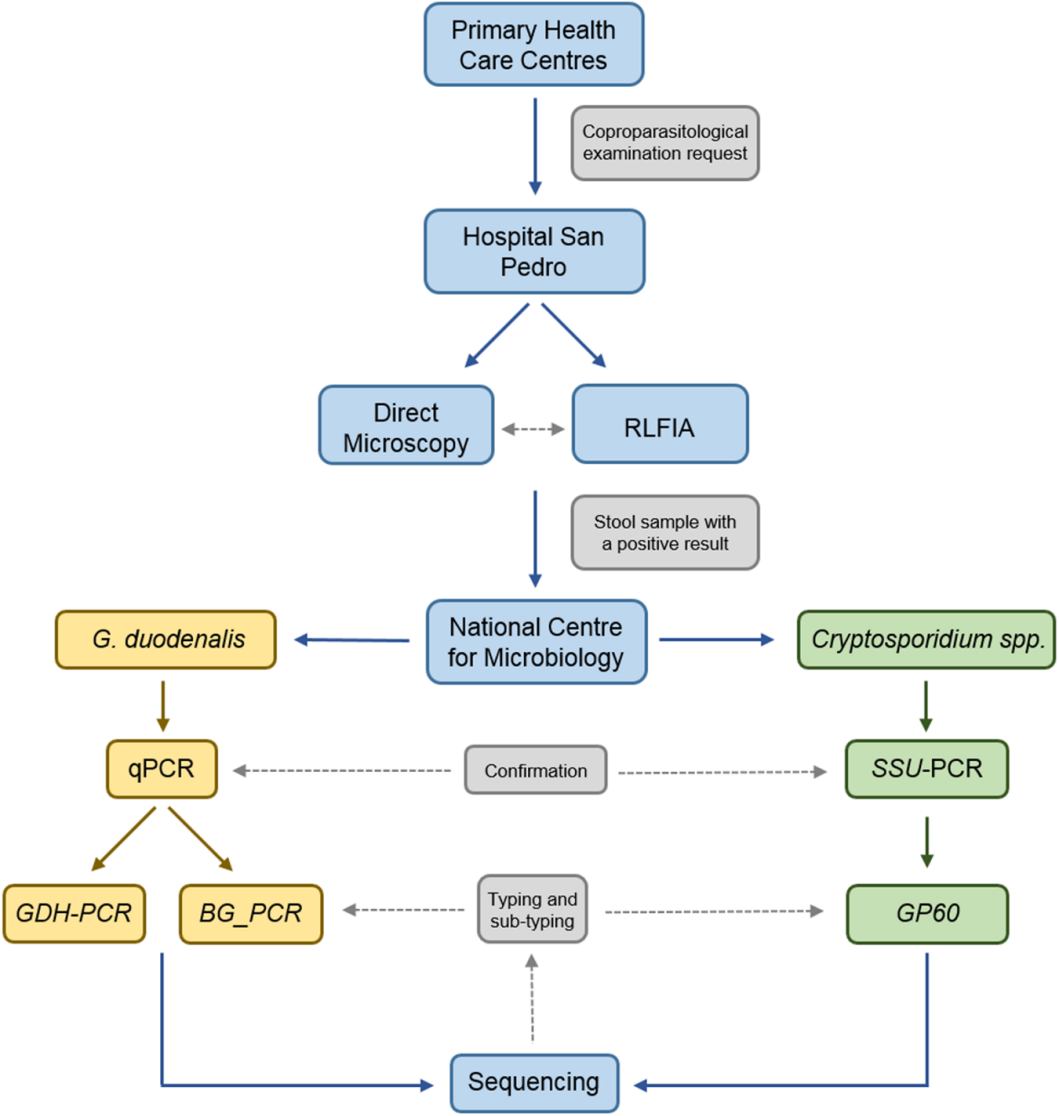
CONSORT flow diagram showing the flow of information, clinical samples, and diagnostic procedures followed in the present study.

In this longitudinal epidemiological survey we investigated the presence of *G*. *duodenalis* and *Cryptosporidium* spp. in outpatients from 28 primary health care centres and inpatients attended at the HSP with a formal request for coproparasitological examination (Fig 1). Detection of parasite (oo)cysts was simultaneously attempted by direct microscopy and a rapid lateral flow immunochromatographic assay (RLFIA). Additionally, both methods were also routinely used to assess the presence of *G*. *duodenalis* or *Cryptosporidium* spp. in all the stool samples received at the Microbiology Laboratory of the HSP from patients of paediatric age (0‒15 years-old) or from patients of older age with clinical (acute or persistent diarrhoea, abdominal pain, cramps, malabsorption, dysbiosis, weight loss, immunosuppression) or epidemiological (recent history of travelling abroad, relatives with gastrointestinal disease) features compatible with giardiosis or cryptosporidiosis. Active search of patients with syndromic symptoms was conducted in a number of cases. None of the patients participating in this study received or were receiving antibiotic treatment with metronidazole, tinidazole, nitazoxanide, paromomicine, furazolidone or albendazole prior to diagnosis. Aliquots of stool specimens that tested positive at microscopy and/or RLFIA were shipped to the Spanish National Centre for Microbiology (Majadahonda) for further molecular analysis (Fig 2). Socio-demographic (gender, age, place of birth) and clinical (symptoms, concomitant infections, general immune status, prescribed treatment) data were also retrieved from the HSP medical records for each individual case with a presumptive or confirmed diagnosis of giardiosis/cryptosporidiosis. The study was conducted from July 2015 to September 2016.

### Direct microscopy

Fresh stool samples were kept at 4°C in the absence of preservative reagents and processed within 2 days of collection following routine diagnostic procedures at HSP. Briefly, a pea-sized amount (equivalent to approximately 1 gram) of faecal material was concentrated using the stool concentration system Midi Parasep SF (Apacor, Wokingham, UK) according to the manufacturer’s instructions. This device included SAF (sodium acetate-acetic acid-formalin) Fixative and Triton X as reagents. The Kinyoun acid-fast stain was specifically used for the detection of *Cryptosporidium* spp. oocyst. One drop (equivalent to approximately 50 μL) of deposit from concentrated material was microscopically examined at 100 magnification, switching to 400 magnification when structures morphologically compatible with *G*. *duodenalis* cysts or *Cryptosporidium* oocysts were suspected.

### Rapid lateral flow immunochromatographic assay (RLFIA)

A one-step rapid lateral flow immunochromatographic assay for the simultaneous detection of *G*. *duodenalis* and *Cryptosporidium* spp. (Simple *Crypto*-*Giardia*, Operon Immuno & Molecular Diagnostics, Zaragoza, Spain) was used according to the manufacturer’s instructions. The assays were conducted at room temperature exclusively on fresh faecal samples. This technique was based on genus-specific monoclonal mouse antibodies directed against *G*. *duodenalis* and *C*. *parvum* antigens allegedly present in all the developmental stages of both pathogens. Claimed diagnostic sensitivities and specificities were in the range of 94‒99% and 79‒99%, respectively, taking ELISA as reference method. The test does not cross-react with faecal samples of patients infected with other enteric parasites including *Entamoeba coli*, *Blastocystis hominis*, *Iodamoeba bütschlii*, *Chilomastix mesnili*, *Endolimax nana*, or *Taenia* spp.

### DNA extraction and purification

Genomic DNA was extracted and purified from a fresh aliquot (~200 mg) of each stool specimen using the QIAamp DNA Stool Mini Kit (QIAGEN, Hilden, Germany) according to the manufacturer’s instructions. DNA isolates (200 μL) were stored at –20°C for further downstream molecular analysis. A water extraction control was routinely included in each sample batch processed.

### Molecular detection of *Giardia duodenalis*

The detection of *G*. *duodenalis* in stool specimens was initially accomplished by a real-time PCR (qPCR) [30] specifically designed to amplify a 62-bp region of the small subunit ribosomal RNA *(SSU* rRNA) gene of the parasite using the primer pair and probe described in S1 Table. Taking advantage of the multi-copy nature of the *SSU* rRNA locus, this method provides enhanced diagnostic sensitivities in large molecular epidemiological surveys. Amplification reaction mixes (25 μL) included 3 μL of genomic DNA, 500 nM of each primer, 200 nM of probe, and TaqMan Gene Expression Master Mix (Applied Biosystems, California, USA). Following the manufacturer´s recommendation, we used a standardised TaqMan amplification protocol consisting on an initial hold step of 2 min at 55°C and 15 min at 95°C followed by 45 cycles of 15 s at 95°C and 1 min at 60°C. Appropriate positive, negative, and inhibition controls were routinely included in each round of qPCR assays. Amplification and detection of parasitic DNA were performed on a Corbett Rotor-Gene 6000 qPCR cycler (Qiagen Corbett, Hilden, Germany). Rotor Gene 6000 Series software version 1.7 was used for data analysis. Fluorescence (510 nm) was measured at the end of the annealing step of each cycle. The ramping of the machine was 10°C/s in every step.

### Molecular characterization of *Giardia duodenalis* isolates

*G*. *duodenalis* isolates that tested positive by qPCR were subsequently analysed using a multi-locus genotyping (MLG) approach based on sequencing data generated by PCR of the genes glutamate dehydrogenase (*GDH*) and ß-giardin (*BG*). A semi-nested PCR protocol targeting a 432-bp fragment of the *GDH* gene was carried out according to [31] with minor modifications. Briefly, both primary and secondary PCR reactions (25 μL) consisted of 5 μL of template DNA, 500 nM of each primer (S1 Table), 2.5 units of MyTAQ DNA polymerase (Bioline GmbH, Luckenwalde, Germany), and 5 μL of MyTAQ Reaction Buffer containing 5 mM dNTPs and 15 mM MgCl_2_. Cycling conditions for PCR amplifications were as follows: 1 cycle of 95°C for 3 min, followed by 35 cycles of 95°C for 30 s, 55°C for 30 s and 72°C for 1 min. A final extension of 72°C for 7 min and a 4°C hold was used.

A nested-PCR protocol targeting a 511-bp partial sequence of the *BG* gene was conducted as described by [32]. Both primary and secondary amplification reactions (25 μL) included 3 μL of template DNA, 400 nM of each primer (S1 Table), 2.5 units of MyTAQTM DNA polymerase (Bioline GmbH), and 5 μL of MyTAQTM Reaction Buffer containing 5 mM dNTPs and 15 mM MgCl_2_. The primary PCR was initiated with a denaturation step of 95°C for 7 min, followed by 35 cycles of 95°C for 30 s, 65°C for 30 s, and 72°C for 1 min with a final extension of 72°C for 7 min. The conditions for the secondary PCR were identical to the primary PCR except that the annealing temperature was 55°C.

PCR reactions were carried out on a 2720 thermal cycler (Applied Biosystems). Laboratory-confirmed positive and negative DNA samples were routinely used as controls and included in each round of PCR. PCR amplicons were visualized on 2% D5 agarose gels (Conda, Madrid, Spain) stained with Pronasafe nucleic acid staining solution (Conda). Positive-PCR products were directly sequenced in both directions using the corresponding internal primer set (S1 Table). DNA sequencing was conducted by capillary electrophoresis using the BigDye Terminator chemistry (Applied Biosystems).

### Molecular detection and characterization of *Cryptosporidium* species

Because the vast majority of human *Cryptosporidium* infections are caused by human-specific *C*. *hominis* and zoonotic *C*. *parvum*, and in an attempt to optimise time, effort, and resources, we adopted a detection and typing scheme primarily based on the amplification and sequence analysis of the gene codifying for the 60 kDa glycoprotein (*GP60*), a highly changeable surface antigen of the parasite which is preferentially utilized in molecular epidemiological investigations [15]. This region is featured by a variable number of tandem repeats of the serine-coding trinucleotide TCA/TCG/TCT at the 5´end of the gene, allowing the categorization of *C*. *hominis* and *C*. *parvum* (among other *Cryptosporidium* species) isolates within distinct *GP60* sub-genotype families or alleles [33]. Faecal samples that initially tested negative at the *GP60* locus were subsequently assessed at the *SSU* rRNA marker in order to confirm the presence of *Cryptosporidium* isolates that were not detectable or typeable at the former gene.

A nested-PCR protocol targeting a 870-pb partial fragment of the *GP60* gene was used according to [34]. Briefly, 3 and 2 μL of template DNA were amplified in the first and second round of PCR, respectively, using 300 nM of each primer (S1 Table). Amplification reaction mixes (50 μL) also contained 2.5 units of MyTAQ DNA polymerase (Bioline GmbH), and 10 μL of MyTAQ Reaction Buffer consisting of 5 mM dNTPs and 15 mM MgCl_2_. Primary cycling conditions were as follow: 5 min at 94°C followed by 35 cycles of 45 s at 94°C, 45 s at 59°C and 1 min at 72°C, with a final extension of 72°C for 10 min. The secondary PCR was similar to that described for the primary PCR step with the exception that the annealing temperature was 50°C.

Finally, a nested PCR targeting a 587-bp fragment of the *SSU* rRNA gene of the parasite was also used [35]. Reaction mixes (50 μL) comprised 3 μL of template DNA, 300 nM of each primer (S1 Table), 2.5 units of MyTAQ DNA polymerase (Bioline GmbH), and 10 μL of MyTAQ Reaction Buffer consisting of 5 mM dNTPs and 15 mM MgCl_2_. Both primary and secondary PCR reactions were carried out as follows: one cycle of 94°C for 3 min, followed by 35 cycles of 94°C for 40 s, 50°C for 40 s and 72°C for 1 min, ending with a final extension of 72°C for 10 min. Agarose gel electrophoresis and DNA sequencing procedures and reagents were as described above for *G*. *duodenalis* isolates.

### Data analyses

Raw sequencing data in both forward and reverse directions were visually inspected using the Chromas Lite version 2.1 sequence analysis program (http://chromaslite.software.informer.com/2.1/). Special attention was paid to the identification of heterozygous sites (double peaks) in the electropherograms. The BLAST tool (http://blast.ncbi.nlm.nih.gov/Blast.cgi) was used to compare nucleotide sequences with sequences deposited in the NCBI. DNA consensus sequences were aligned to reference sequences using ClustalW in MEGA version 6.0 (http://www.megasoftware.net/) to determine *G*. *duodenalis* assemblages and sub-assemblages, and *Cryptosporidium* species and sub-genotypes. Phylogenetic analyses, based on the Neighbour-Joining method, were performed using the same software [36]. Sequences including heterozygous (di-nucleotide) sites were excluded from the analyses in order to avoid distorting the topology of the phylogenetic trees.

The sequences obtained in this study have been deposited in GenBank under accession numbers KY499033 to KY499054 (*G*. *duodenalis*) and KY499055 to KY499059 (*Cryptosporidium* spp.).

## Results

Stool samples were obtained from 209 patients referred to or attended at the HSP that tested positive for *G*. *duodenalis* (N = 106) or *Cryptosporidium* spp. (N = 103) by direct microscopy and/or RLFIA during the period of study. Of them, 88% (184/209) corresponded to single specimens and the remaining 12% (25/209) to pooled specimens of consecutively collected stool samples (S2 Table).

### Analysis of socio-demographic variables

Table 1 shows the socio-demographic variables of the patients found infected with giardiosis or cryptosporidiosis by any of the diagnostic methods used in the present study. The male/female ratios were 0.71 and 1.19, respectively. Infants and children between 6 months and 12 years of age were particularly susceptible to these diseases, with up to 60% of the detected *Cryptosporidium* infections occurring in the age group 0–4 years of age. Although adult individuals in the age group 26–50 also represented a substantial proportion of the cases of giardiosis, only sporadic cases of cryptosporidiosis were observed in subject older than 25 years. Interestingly, *G*. *duodenalis* infections were more frequently detected in females (59%) than in males (42%). The majority of the documented cases of giardiosis and cryptosporidiosis were nationals (78%–89%) with no (88%–98%) recent history of travelling abroad.

**Table 1:** Socio-demographic variables of infected individuals with human giardiosis and cryptosporidiosis attended at the San Pedro Hospital. Logroño (La Rioja), 2015–2016.

	*Giardia duodenalis*		*Cryptosporidium* spp.	
	Cases N = 106	%	Cases N = 103	%
**Gender**				
Male	44	41.5	56	54.4
Female	62	58.5	47	45.6
**Age group (years)**				
0–4	35	33.0	61	59.2
5–12	42	39.6	34	33.0
13–25	6	5.7	5	4.9
26–50	18	17.0	1	1.0
>50	5	4.7	2	1.9
**Country of origin**				
Spain	83	78.3	92	89.3
Other European countries	1	0.9	6	5.8
Africa	10	9.4	3	2.9
America	5	4.7	1	1.0
Asia	7	6.6	1	1.0
**Travelling abroad**				
No	93	87.7	101	98.1
Yes	13	12.3	2	1.9

### Analysis of clinical variables

Table 2 summarizes the main clinical variables recorded in patients with giardiosis and cryptosporidiosis in the present survey. *G*. *duodenalis* and *Cryptosporidium* spp. infections were asymptomatic in 18% and 3% of the cases, respectively. The clinical manifestations more commonly associated with giardiosis or cryptosporidiosis were acute (43%–77%) or chronic (7%–20%) diarrhoea and abdominal pain (21%–22%). Nausea/vomit was also reported by a substantial proportion (20%) of patients with *Cryptosporidium* spp. infections, but was less frequent in individuals with giardiosis (6%). Most infections cases (96%–98%) were detected in immunocompetent subjects, although two patients with giardiosis and two with cryptosporidiosis were affected by malformation and nephrotic syndromes, respectively. *Cryptosporidium* infections were found in two additional patients diagnosed with mosaicism and tetralogy of Fallot, respectively. Patients with giardiosis and cryptosporidiosis also harboured with relative frequency a number of pathogenic and commensal microorganisms including bacteria, protozoa, or viruses, being the most frequent those members of the bacterial genus *Campylobacter*. Regarding pharmacological treatment, 84% of the patients with giardiosis were administered with metronidazole orally (adults: 250 mg every 8 hours; children: 15 mg/kg/day every 8 hours) for 5 consecutive days, whereas the remaining cases remained untreated. Patients affected by cryptosporidiosis received no specific treatment.

**Table 2:** Clinical variables of infected individuals with giardiosis or cryptosporidiosis attended at the San Pedro Hospital, Logroño (La Rioja), 2015–2016.

	*Giardia duodenalis*		*Cryptosporidium* spp.	
	Cases N = 106	%	Cases N = 103	%
**Clinical symptoms**				
Asymptomatic	19	17.9	3	2.9
Acute diarrhoea	46	43.4	79	76.7
Chronic diarrhoea	21	19.8	7	6.8
Abdominal pain	22	20.8	23	22.3
Nausea/vomit	6	5.7	21	20.4
Constipation	2	1.9	0	0.0
Flatulence	1	0.9	0	0.0
Anorexy	0	0.0	3	2.9
Rectal bleeding	1	0.9	0	0.0
Pruritus ani	0	0.0	1	1.0
Eosinophilia	1	0.9	0	0.0
Atopic dermatitis	1	0.9	0	0.0
Fever	0	0.0	1	1.0
Failure to thrive	3	2.8	6	5.8
Cognitive impairment	1	0.9	0	0.0
**Immune debilitating conditions**				
None	104	98.1	99	96.1
Malformation syndrome	1	0.9	1	1.0
Nephrotic syndrome	1	0.9	1	1.0
Mosaicism	0	0.0	1	1.0
Tetralogy of Fallot; lymphedema- distichiasis syndrome	0	0.0	1	1.0
**Co-infections**				
None	85	80.2	89	86.4
*Blastocystis hominis*	3	2.8	0	0.0
*Crytosporidium* spp.	6	5.7	NA	NA
*Giardia duodenalis*	NA	NA	3	2.9
*Entamoeba coli*	2	1.9	0	0.0
*Iodamoeba butschlii*	2	1.9	0	0.0
*Hymenolepis nana*	1	0.9	0	0.0
*Aeromonas hidrophyla*	0	0.0	1	1.0
*Campylobacter* spp.	7	6.6	7	6.7
*Salmonella* spp.	2	1.9	1	1.0
Rotavirus	0	0.0	2	1.9
**Treatment**				
Metronidazole	89	84.0	NA	NA
None	17	16.0	NA	NA

NA: not applicable.

### Comparison of the diagnostic methods used for the detection of *G*. *duodenalis* and *Cryptosporidium* spp.

Direct microscopy and RLFIA produced coincidental positive results for the detection of *G*. *duodenalis* and *Cryptosporidium* spp. in 71% (75/106) and 92% (95/103) of the studied specimens, respectively (Table 3). The presence of both pathogens was confirmed by PCR-based methods in 92% and 82%, respectively, of the samples that previously tested positive by direct microscopy and RLFIA. In this regard, *G*. *duodenalis*-positive isolates by qPCR generated cycle threshold (Ct) values ranging from 19.2 to 38.6 (mean: 30.3; SD: 11.4). Interestingly, whereas most (85%–100%) of the direct microscopy-positive, but RLFIA-negative, results for *G*. *duodenalis* and *Cryptosporidium* spp. could be confirmed by PCR, the opposite (direct microscopy-negative, but RLFIA-positive results) was true only in 50% and 17% of the cases, respectively. Based on these figures the false-positive rate of RLFIA was estimated at 8.5% (9/106) for *G*. *duodenalis* and 4.9% (5/103) for *Cryptosporidium* spp.

**Table 3:** Concordance of the results obtained by direct microscopy (DM) and rapid lateral flow immunochromatographic assay (RLFIA) for the detection of *G*. *duodenalis* and *Cryptosporidium* spp. in the present study. Numbers and percentages of samples confirmed by PCR-based methods are also indicated.

	*G*. *duodenalis* (N = 106)			*Cryptosporidium* spp. (N = 103)		
	No. samples	Percentage	No. PCR-confirmed samples (%)	No. samples	Percentage	No. PCR-confirmed samples (%)
**DM (+) and RLFIA (+)**	75	70.7	70 (66.0)	95	92.2	78 (75.7)
**DM (+) and RLFIA (-)**	13	12.3	11 (10.4)	2	1.9	2 (1.9)
**DM (-) and RLFIA (+)**	18	17.0	9 (8.5)	6	5.8	1 (1.0)
**Total**	106	100		103	100	

### Detection and genotyping of *G*. *duodenalis* isolates

Out of the 90 *G*. *duodenalis*-positive isolates by qPCR (Table 3), amplification success rates for the *GDH*-PCR and *BG*-PCR assays were 17.8% (16/90) and 4.4% (4/90), respectively. Multi-locus sequencing data at the *GDH* and *BG* loci were only available for four *G*. *duodenalis* isolates (S2 Table). The limited sensitivity of the *GDH*-PCR and *BG*-PCR assays was a direct consequence of the low concentration of parasitic DNA available as template in their respective amplification reactions, as demonstrated by the fact that only samples with qPCR Ct values lower than 30 were consistently characterized at these markers. This problem was further exacerbated when considering that samples with Ct values ≥ 30 constituted more than half (49/90) of the cases confirmed by qPCR (S2 Table).

Table 4 summarizes the frequency and molecular diversity of the *G*. *duodenalis* isolates fully sub-genotyped in the present study. Sequence alignment analyses of the 14 *GDH* amplicons characterized revealed the presence of sub-assemblages AII (57%, 8/14), BIII (7%, 1/14), and BIV (36%, 5/14). Two additional isolates were assigned to BIII (S2 Table), but poor quality sequencing data prevented accurate analysis at the nucleotide level. Out of the eight AII sequences, six showed 100% identity with a common variant (L40510) of this particular sub-assemblage, one (KY499034) corresponded to a novel isolate with two single nucleotide polymorphisms (SNPs) inducing amino-acid changes at the protein level, and the remaining one (KY499035) presented an internal stop codon resulting in a non-functional protein (Table 4). Novel sequences were also identified within members of sub-assemblages BIII and BIV. A high degree of genetic variation, ranging from 4 to 6 SNPs, was observed in most BIV sequences, including the presence of double peaks in the corresponding electropherogram (KY499038) and a number of mutations inducing changes in the affected amino-acid sequences.

**Table 4:** Diversity, frequency, and molecular features of *Giardia duodenalis* in clinical human isolates, San Pedro Hospital, Logroño (La Rioja), 2015–2016.

Assemblage	Sub-assemblage	No. isolates	Locus	Reference sequence	Stretch	SNPs	GenBank accession No.
A	AII	6	*GDH*	L40510	88‒470	None	KY499033
		1	*GDH*	L40510	88‒470	T139C^a^, T430C^b^	KY499034
		1	*GDH*	L40510	88‒470	A223T^c^	KY499035
	BIII	1	*GDH*	AF069059	75‒460	G306A, C309T, G311A^d^, C336T	KY499036
	BIV	2	*GDH*	L40508	88‒453	None	KY499037
		1	*GDH*	L40508	88‒453	T183C, T290Y^e^, T387C, C396T, C423T	KY499038
		1	*GDH*	L40508	88‒453	T183C, T387C, C396T, C423T	KY499039
		1	*GDH*	L40508	88‒453	T183C, T355C^f^, T387C, G390A, C396T, C423T	KY499040
	AII	1	*BG*	AY072723	99‒594	None	KY499041
	AIII	1	*BG*	AY072724	99‒594	None	KY499042
B	‒	1	*BG*	AY072727	99‒593	A183G, C309Y, T519Y, C564Y	KY499043

GenBank accession numbers for representative sequences were provided. Novel assemblages/sub-assemblages were shown underlined. Point mutations inducing amino acid substitutions or stop codons were highlighted as superscript numbers indicating the amino acid change. SNP: single nucleotide polymorphism.

^a^p.Phe47Leu.

^b^p.Phe144Leu.

^c^p.Arg75* (stop codon).

^d^p.Gly104Asp.

^e^p.Val97Ala.

^f^p.Phe118Leu.

Sequence alignment analysis of the three amplicons successfully genotyped at the *BG* locus allowed their allocation to the sub-assemblages AII and AIII, and the assemblage B, respectively (Table 4). An additional isolate was positively assigned to B but sub-optimal sequencing data precluded detailed studies at the nucleotide level (S2 Table). No mixed assemblage A and B infections were detected. A discordant genotype result AII/AIII was identified in a single isolate at the *GDH* and *BG* loci.

**Fig 3:**
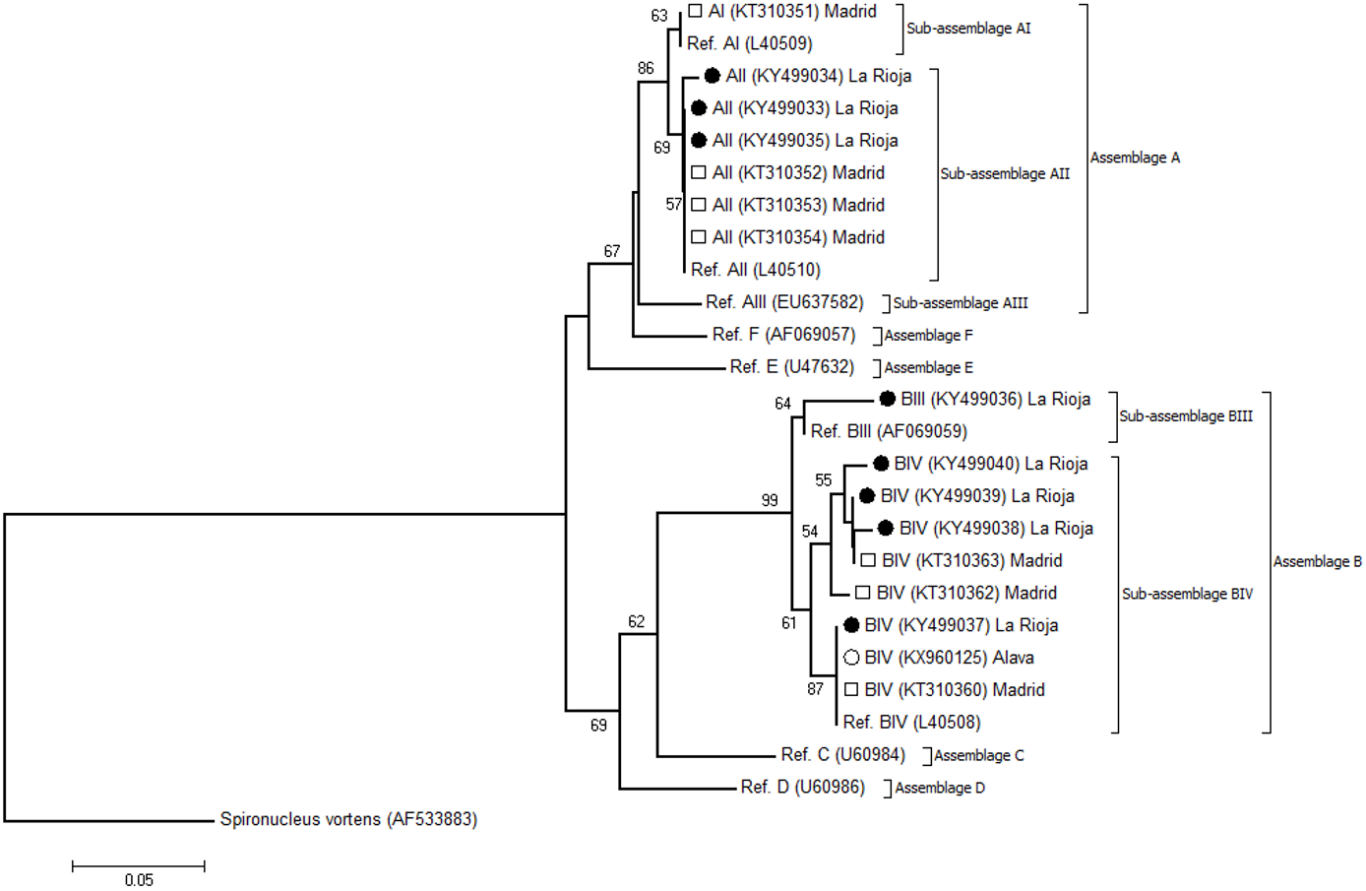
Evolutionary relationships among *G*. *duodenalis* sub-assemblages at the *GDH* locus inferred by a Neighbor-Joining analysis of the nucleotide sequence covering a 383-bp region (positions 88 to 470 of GenBank accession number L40508) of the gene. The percentage of replicate trees in which the associated taxa clustered together in the bootstrap test (1,000 iterations) is indicated next to the branches. Bootstrap values lower than 50% were not displayed. The evolutionary distances were computed using the Kimura 2-parameter method. The rate variation among sites was modelled with a gamma distribution (shape parameter = 2). Filled circles represent AII, BIII, and BIV sequences generated in this study. Open circles indicate *G*. *duodenalis* sequences of human origin previously reported in other Spanish regions that were included in the analysis for comparative purposes. *Spironucleus vortens* was used as outgroup taxa.

Fig 3 shows the phylogenetic relationship among *G*. *duodenalis* sub-assemblages at the *GDH* marker. The tree was constructed by the Neighbour-Joining method using the unambiguous (homozygous) sequences generated in the present study and appropriate reference sequences downloaded from the NCBI database. The topology of the produced phylogenetic tree clearly shows that isolates belonging to sub-assemblages AII, BIII, and BIV clustered together in well-defined, distinct groups.

### Detection and genotyping of *Cryptosporidium* spp. isolates

Out of the 103 isolates from patients diagnosed with cryptosporidiosis by DM and/or RLFIA, 65% (67/103) were directly sub-genotyped at the *GP60* locus. All 36 isolates that initially tested negative at the *GP60*-PCR were subsequently re-assessed at the *SSU* marker, allowing the confirmation of the presence of *Cryptosporidium* spp. in 14 additional isolates (S2 Table). Therefore, the presence of the parasite was confirmed by any of the PCR methods used in 79% (81/103) of the cases (Table 3).

Sequence alignment analyses of both *GP60* and *SSU* isolates demonstrated that *C*. *hominis* (81%, 66/81) and *C*. *parvum* (19%, 15/81) were the only *Cryptosporidium* species found in the population under study. Obtained molecular data revealed the presence of *GP60* sub-genotype families Ib (73%, 59/81) within *C*. *hominis*, and IIa (7%, 6/81) and IId (2%, 2/81), within *C*. *parvum* (Table 5). Additionally, seven isolates were assigned to *C*. *hominis* (9%, 7/81) and another seven to *C*. *parvum* (9%, 7/81) at the *SSU* marker. IbA10G2 was the *Cryptosporidium* sub-genotype more abundantly circulating, being identified in 88% (59/67) of the isolates, including five novel genotypic variants. Sub-genotypes including IIaA15G2R1 (6%, 4/67), IIdA17G1 (3%, 2/67), IIaA14G2R1 (1.5%, 1/67), and IIdA16G2R1 (1.5%, 1/67), some of them constituting novel isolates of the parasite, were far less represented (Table 5). A variable number of polymorphic sites including the presence of double peaks (KY499048), point mutations inducing stop codons (KY499047 and KY499050), insertions (KY499052), and deletions (KY499056 to KY499059) were observed in some of the *GP60* and *SSU* sequence analysed (Table 5), revealing a moderate degree of genetic variability. This was particularly true for isolates belonging to *C*. *parvum*.

**Table 5:** Diversity, frequency, and main molecular features of *Cryptosporidium* isolates at the *GP60* and *SSU* rRNA loci in clinical samples, San Pedro Hospital, Logroño (La Rioja), 2015–2016.

Marker	Species	Family	Sub-genotype	No. isolates	Reference sequence	Stretch	SNPs	GenBank accession No.
*GP60*	*C*. *hominis*	Ib	IbA10G2	54	AY262031	24−857	None	KY499044
				1	AY262031	24−857	G216T	KY499045
				1	AY262031	24−857	A327G, A756G	KY499046
				1	AY262031	24−857	T425A^a^	KY499047
				1	AY262031	24−857	C497Y^b^, C581Y^c^	KY499048
				1	AY262031	24−857	C579M	KY499049
	*C*. *parvum*	IIa	IIaA14G2R1	1	JF727773	6−858	T37C^d^, C270T^e^, T480G^f^, A486G, C529T^g^	KY499050
			IIaA15G2R1	4	AY262034	43−872	None	KY499051
			IIaA16G2R1	1	AY262034	45−872	146_147insATC	KY499052
			IIdA17G1	1	LT556065	9−830	G277A^h^	KY499053
				1	LT556065	9−830	G277A^h^, T279A	KY499054
*SSU*	*C*. *hominis*	‒	‒	7	AF108865	567−978	None	KY499055
	*C*. *parvum*	‒	‒	4	AF112571	539−1,026	A646G, T649G, 686_689delTAAT, T693A	KY499056
	*C*. *parvum*	‒	‒	1	AF112571	533−1,030	A646G, T649G, 686_689delTAAT, T693A, C751A	KY499057
	*C*. *parvum*	‒	‒	1	AF112571	535−1,026	A646G, 647_649delATT, T663C, 686_689delTAAT	KY499058
	*C*. *parvum*	‒	‒	1	AF112571	573−997	A646G, T649G, 686_689delTAAT, C795Y	KY499059

GenBank accession numbers of representative sequences were provided. Novel genotypes were shown underlined. Point mutations inducing amino acid substitutions or stop codons were highlighted as superscript numbers indicating the amino acid change. SNP: single nucleotide polymorphism.

^a^p.Leu142*.

^b^p.Ser166Phe.

^c^p.Ser194Phe.

^d^p.Leu12Ser.

^e^p.Gln90*.

^f^p.Tyr160Asp.

^g^p. Ser176Phe.

^h^p.Ala93Thr.

**Fig 4:**
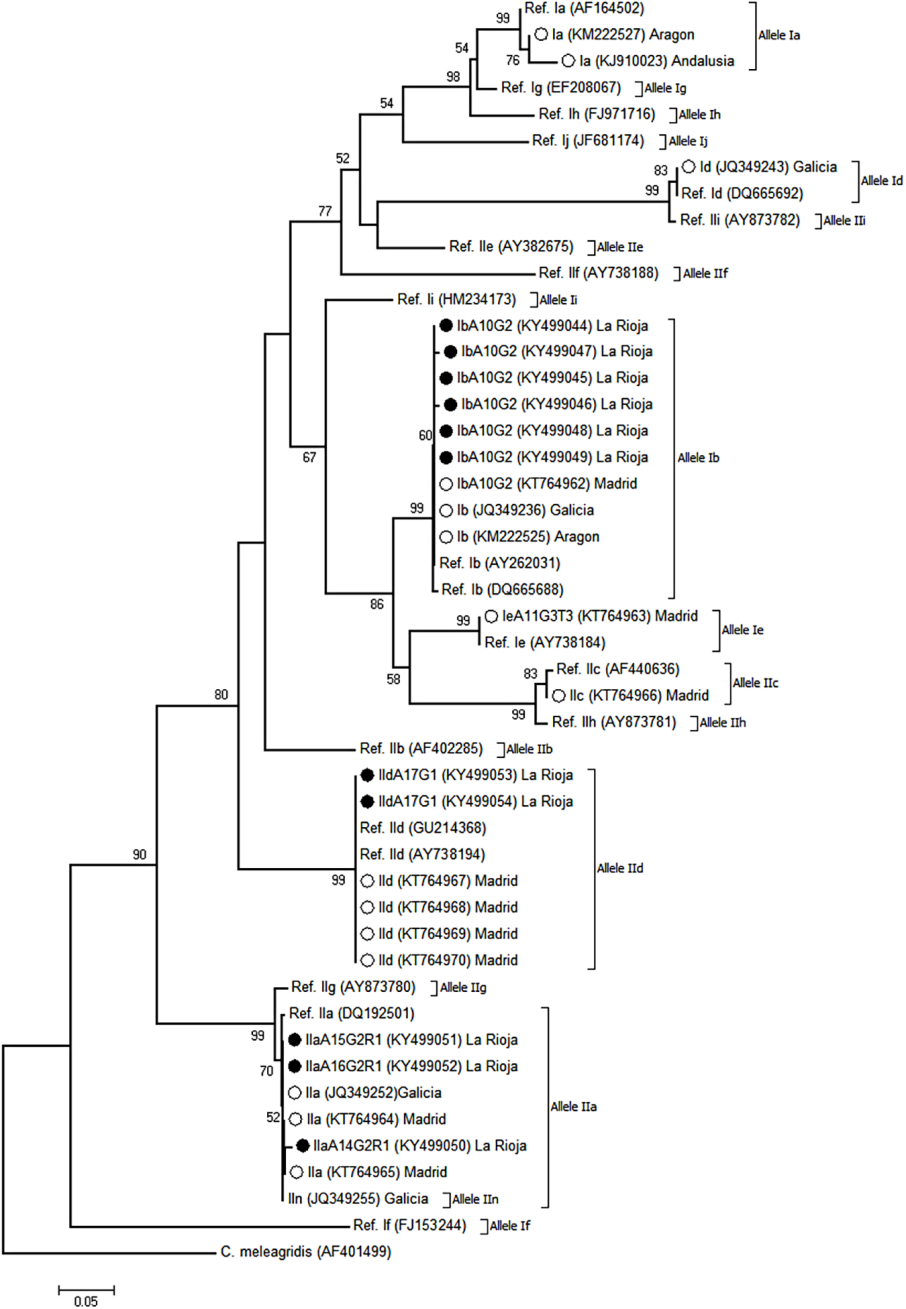
Evolutionary relationships among *C*. *hominis* and *C*. *parvum* sub-genotypes at the *GP60* locus inferred by a Neighbor-Joining analysis of the nucleotide sequence covering a 810-bp region (positions 47 to 856 of GenBank accession number AY262031) of the gene. The percentage of replicate trees in which the associated taxa clustered together in the bootstrap test (1,000 iterations) is indicated next to the branches. Bootstrap values lower than 50% were not displayed. The evolutionary distances were computed using the Kimura 2-parameter method. The rate variation among sites was modelled with a gamma distribution (shape parameter = 2). Filled circles represent Ib, IIa, and IId sequences generated in this study. Open circles indicate *Cryptosporidium s*equences of human origin previously reported in other Spanish regions that were included in the analysis for comparative purposes. *C*. *meleagridis* was used as outgroup taxa.

Fig 4 shows the phylogenetic relationship among *C*. *hominis* and *C*. *parvum* sub-genotypes at the *GP60* locus produced by the Neighbour-Joining method. As in the case of Fig 3, only unambiguous (homozygous) sequences were used. As expected, all the isolates assigned to the *Cryptosporidium* sub-genotype families Ib, IIa, and IId in the present study were located in well-defined clusters with their corresponding reference sequences.

### Temporal and geographical distribution of *G*. *duodenalis* and *Cryptosporidium* spp. infections

No obvious seasonal pattern was observed for the human cases of giardiosis recorded during the 15-month period of study. Similarly, the 16 *G*. *duodenalis* isolates genotyped at the sub-assemblage level were homogeneously distributed through both urban and rural areas of the surveyed region (Fig 1). Taken together, these epidemiological and molecular data seem to indicate that transmission of human giardiosis in La Rioja occur primarily through occasional infections rather than through water-borne or food-borne outbreaks.

In contrast, human cryptosporidiosis displayed a marked bimodal seasonal distribution pattern during the second half of 2015, with a large peak predominantly involving the IbA10G2 sub-genotype of the parasite occurring in August and a smaller one in middle autumn. Interestingly, this trend was not observed in the following year, when fare less cases of *Cryptosporidium* infections were documented (Fig 1). As in the case of *G*. *duodenalis* infections, no apparent differences in the geographical distribution of *Cryptosporidium* cases were observed in La Rioja, although three quarters of the *C*. *parvum* infections were detected in the capital city, Logroño.

## Discussion

Human giardiasis and cryptosporidiosis are among the 52 communicable diseases and health issues for which surveillance is mandatory in the European Union (EU). Only in 2012 a total of 16,368 confirmed cases of giardiasis (5.4 cases per 10^−5^ population) and 9,581 cases of cryptosporidiosis (3.2 cases per 10^−5^ population) were officially reported in the EU [14]. In Spain, giardiasis and cryptosporidiosis were declared compulsory notifiable diseases as late as March 2015 [37], meaning that the actual epidemiological situation of both infections remains largely unknown. Additionally, robust genotyping data on the frequency and genetic diversity of *G*. *duodenalis* and *Cryptosporidium* spp. are restricted to few large molecular surveys conducted mainly in paediatric populations of symptomatic [18,19,22–24,26] and asymptomatic [20,21] individuals.

As anticipated, human giardiosis and cryptosporidiosis in La Rioja affect primarily infants and children between 6 months and 12 years of age, corroborating previous results from other Spanish regions such as Barcelona [26], Madrid [19,20,23], Galicia [24], and Zaragossa and Leon [22]. Notably, a significant proportion of the *G*. *duodenalis* and *Cryptosporidium* spp. infections described in the present survey were accidentally detected during routine microscopic examination. Worryingly, a number of the diagnosed cases were considered by general practitioners unworthy of treatment and/or follow up. This situation evidences the necessity of increasing the awareness among health care providers and explains, at least partially, the severe under-reporting of these infections in Spain and other European countries [15].

In Spain, giardiosis, and to a lesser extent cryptosporidiosis, have been identified as significant contributors to the burden of imported infectious diseases associated to immigrants [38] and travellers from or to endemic areas [39,40]. However, the *G*. *duodenalis* and *Cryptosporidium* spp. infections reported here very likely represent autochthonous cases, as most of the patients investigated were nationals with no recent history of travelling abroad. The same conclusion was reached in similar molecular epidemiological surveys carried out in the Madrid area [19,23]. As expected, acute diarrhoea, abdominal pain and nausea/vomit were the clinical manifestations more frequently identified in the symptomatic population under study. Infections by *G*. *duodenalis* and *Cryptosporidium* spp. were primarily found in immunocompetent individuals, whereas co-infections with other infectious pathogens were only occasionally detected. Far more worrying was the finding that a number of our paediatric cases developed failure to thrive and cognitive impairment, conditions previously thought to be present only in malnourished children with giardiosis/cryptosporidiosis in developing countries [41]. This fact seems to suggest that the socioeconomic impact of giardiosis and cryptosporidiosis in Spain, and very likely in other developed countries, may be much higher than initially anticipated.

Regarding their seasonal patterns, *G*. *duodenalis* cases were consistently detected through the whole period of study without clear incidence peaks, suggesting that the spread of the infection should be mainly a result of anthroponotic risk factors and transmission as previously suggested [42]. In contrast, a marked annual and inter-annual variation in *Cryptosporidium* cases was observed. Firstly, most *Cryptosporidium* infections concentrated in 2015, whereas only sporadic cases were detected the following year. Secondly, only 2015 cases followed the bi-modal distribution with a prominent peak in late summer primarily attributed to *C*. *hominis* IbA10G2 and a smaller one in middle autumn typically described in other European countries [14]. This unexpected, highly variable intra-annual distribution of cryptosporidiosis may be linked to previous surveillance findings reporting an unprecedented 2 to 5-fold increase in the number of *Cryptosporidium* cases in the Netherlands, the United Kingdom, and Germany during the summer months of 2012. Subsequent epidemiological analyses into the cause of that rise did not reveal a clear common source [43], and the possibility of an outbreak was considered unlikely [44]. On the other hand, summer peaks in cryptosporidiosis incidence have been previously linked to host related factors such as recreational water use [42], travelling abroad [43], and seasonal contact with livestock [45], but none of these factors by themselves seem to provide a reasonable explanation of the findings shown in the present study. It is noteworthy that, in the Autonomous region of Galicia (north-west Spain), it was *C*. *parvum* and not *C*. *hominis* the main *Cryptosporidium* species detected in summer peaks during the period 2000–2008, particularly in rural areas [24]. This finding seems to indicate that human cryptosporidiosis cases in that particular region may have a zoonotic source.

As in other European countries, microscopic examination remains the method of choice for the detection of *G*. *duodenalis* and *Cryptosporidium* spp. in most clinical and microbiological laboratories in Spain [46], although RLFIA is rapidly gaining acceptance as diagnostic tool due to simplicity of use and short test time. However, it is important to bear in mind that the diagnostic performance of RLFIA may be hampered by sensitivity [47], specificity [48], and reproducibility [49] issues. In our hands, the RLFIA used here delivered false-positive rates of 8.5% for *G*. *duodenalis* and 4.9% for *Cryptosporidium* spp., respectively, figures well in the range of those (5.2%-9.8%) reported by our research group in previous surveys using similar devices [19,23]. RLFIA-positive results in hospital settings should, therefore, be cautiously interpreted in conjunction with clinical and epidemiological parameters and may require in certain cases confirmation by more specific methods such as PCR or direct immunofluorescence assay.

Remarkably, our genotyping and sub-genotyping results revealed unexpectedly low amplification rates at the *GDH* and *BG* loci (18% vs. 4%) of the *G*. *duodenalis*-positive isolates previously identified by qPCR. The performances of the *GDH*- and *BG*-PCRs carried out here were comparatively lower than those previously achieved by our research group using identical DNA extraction, purification, and amplification protocols in isolates from clinical (61% vs 47%) and general (34% vs 25%) mostly paediatric populations in Spain [19] and Ethiopia [50]. Although the amplification efficiency of genomic nucleic acids isolated from faecal material are known to be influenced by poor quality or low starting concentrations of DNA, the copy number of the targeted gene, and the primer set design [50,51], at present we cannot provide a clear explanation for this phenomenon. Interestingly, AII was the *G*. *duodenalis* sub-assemblage more prevalent in the surveyed human population, being identified in 44% of the genotyped isolates. In contrast, molecular data from other Spanish regions found BIV as the most frequent sub-assemblage in the Madrid area [19] and Zaragossa and Leon [22]. Assemblage B is also responsible of two thirds of the human cases of giardiasis documented in Europe [8]. This finding demonstrates that the distribution pattern of *G*. *duodenalis* assemblages/sub-assemblages vary across geographical areas probably as a result of multifactorial causes including transmission pathways, general prevalence, infection pressure, human population at risk, human behavioural practices, and geo-climatic factors. It is also well-stablished that a marked host distribution exists within assemblage A, with human isolates belonging to sub-assemblages AI and AII and animal isolates belonging to AI and AIII [8,52]. This fact, together with the absence of animal-specific assemblages C-F strongly suggest that transmission of human giardiosis in La Rioja must be predominantly anthroponotic in origin, as previously proposed in other Spanish regions [19,53].

Our molecular data also confirmed the higher degree of genetic variability within B, but not A, *G*. *duodenalis* assemblages commonly observed in similar studies [18,19,54,55]. Although potentially biased by the relatively low number of isolates of the parasite characterised at the nucleotide level, very few B sequences revealed polymorphic (double peaks) sites during chromatogram inspection. Additionally, discordant genotyping results were only observed in a single *G*. *duodenalis* isolate, whereas no co-infections involving different intra- or inter-assemblage genetic variants of the parasite were detected. Taken together, these data are in agreement with the clonal population structure of *Giardia* (an organism assumed to be primitively asexual) described in some population genetic surveys [18,56]. Although these investigations concluded that recombination between *G*. *duodenalis* assemblages is either very rare or absent, the asexual nature of the parasite has been increasingly challenged by mounting evidence demonstrating the exchange of genetic material within and between assemblages of *G*. *duodenalis* isolates of human origin [57–59]. Consistent with this hypothesis, we recently reported elevated numbers of heterozygous (double peaks) sites within B sequences and high rates of discordant AII/AIII or BIII/BIV genotyping results in symptomatic individuals with giardiosis from the Madrid area, both findings being compatible with intra-assemblage recombination events [19]. Definitively more research is needed to conclusively demonstrate the existence of sexual reproduction in *Giardia*.

Regarding *Cryptosporidium*, *C*. *hominis* was responsible for most (81%) of the human cryptosporidiosis cases reported in La Rioja, a slightly lower infection rate compared with those (88%-93%) previously documented in other Spanish areas including Madrid [19], Zaragossa [25], and Barcelona [26]. Interestingly, a *C*. *parvum* prevalence of 34%, more than 3-fold higher than national average, has been recorded in Galicia [24]. Because livestock farming represent an important economic sector in that region, it was suspected that a large proportion of the human cryptosporidiosis cases by *C*. *parvum* had a zoonotic origin [24]. Also in line with the results obtained in previous national [19, 24–26] and international [9,10] molecular epidemiological surveys, *C*. *hominis* IbA10G2 and *C*. *parvum* IIaA15G2R1 were the *Cryptosporidium GP60* sub-genotypes more frequently associated to human cryptosporidiosis in La Rioja. Interestingly, five novel genetic variants involving 1‒2 SNPs were detected within the Ib10G2 sub-genotype. In this regard, it is worth mentioning that recent population genetic analyses have evidenced the exclusive occurrence of genetic recombination in the virulent *C*. *hominis* subtypes IbA10G2 [60,61] and IaA28R4 [61,62], especially around the putative virulence determinant *GP60* region. These findings suggest that genetic recombination may act as an evolutionary agent and driving force in the emergence of virulent *C*. *hominis* subtypes. In the present study a novel *C*. *parvum* IIdA17G1 sub-genotype was confirmed in a single human isolate. Human infections by this particular genetic variant have been occasionally reported in Italy (KU852708), Romania (LT556065), and UK (HQ149040), although cases by other members of the IId family have also been documented in other European countries [15]. In Spain IId sub-genotyped have been identified in lambs [63,64], goats [63], and pre-weaned calves [64], providing evidence of the zoonotic potential of this *C*. *parvum* genetic variant.

## Conclusions

As in other Spanish regions *G*. *duodenalis* and *Cryptosporidium* spp. are important causes of morbidity associated to gastrointestinal illness in individuals seeking medical attention in La Rioja. Our data reflect that both diseases are generally under-diagnosed and their severity and public health impact under-estimated and misjudged in some instances. Our molecular and genotyping data seem to indicate that transmission of giardiosis and cryptosporidiosis in this Spanish region is primarily anthroponotic, although an undetermined number of *C*. *parvum* infection may have a zoonotic origin. We believe that data presented here on the geographical distribution, seasonal patterns, frequency and diversity of genotypes of *G*. *duodenalis* and *Cryptosporidium* spp. constitute a relevant contribution to our current knowledge on the epidemiology and transmission dynamics of these protozoan pathogens in Spain.

## Supporting information

S1 Table Oligonucleotides used for the molecular identification and characterization of *Giardia duodenalis* and *Cryptosporidium* spp. in the present study. (DOCX)

S2 Table Complete data set including the sociodemographic and clinical variables considered in the different analyses conducted and the diagnostic results obtained for the detection and molecular characterization of *Giardia duodenalis* and *Cryptosporidium* spp. in the present study. (XLS)
