# Supplementary material for: Molecular diversity and frequency of the diarrheagenic enteric protozoan Giardia duodenalis and Cryptosporidium spp. in a hospital setting in Northern Spain
Source: PLoS One. 2017 Jun 15;12(6):e0178575. doi: 10.1371/journal.pone.0178575 (PMC5472271; doi:10.1371/journal.pone.0178575)
Supplement: S1 Table — (DOCX) [file pone.0178575.s001.docx]

**S1 Table**

Oligonucleotides used for the molecular identification and characterization of *Giardia duodenalis* and *Cryptosporidium* spp. in this study.

| Target organism | Locus | Oligonucleotide | Sequence (5´–3´) | Reference |
| --- | --- | --- | --- | --- |
| *Giardia duodenalis* | *SSU* rRNA | Probe | FAM–CCCGCGGCGGTCCCTGCTAG–BHQ1 | [30] |
|  |  | Gd-80F | GACGGCTCAGGACAACGGTT | [30] |
|  |  | Gd-127R | TTGCCAGCGGTGTCCG | [30] |
|  | *GDH* | GDHeF | TCAACGTYAAYCGYGGYTTCCGT | [31] |
|  |  | GDHiF | CAGTACACCTCYGCTCTCGG | [31] |
|  |  | GDHiR | GTTRTCCTTGCACATCTCC | [31] |
|  | *BG* | G7-F | AAGCCCGACGACCTCACCCGCAGTGC | [32] |
|  | *BG* | G759-R | GAGGCCGCCCTGGATCTTCGAGACGAC | [32] |
|  | *BG* | G99-F | GAACGAACGAGATCGAGGTCCG | [32] |
|  | *BG* | G609-R | CTCGACGAGCTTCGTGTT | [32] |
| *Cryptosporidium* spp. | *GP60* | AL-3531 | ATAGTCTCCGCTGTATTC | [34] |
|  |  | AL-3535 | GGAAGGAACGATGTATCT | [34] |
|  |  | AL-3532 | TCCGCTGTATTCTCAGCC | [34] |
|  |  | AL-3534 | GCAGAGGAACCAGCATC | [34] |
|  | *SSU* rRNA | CR-P1 | CAGGGAGGTAGTGACAAGAA | [35] |
|  |  | CR-P2 | TCAGCCTTGCGACCATACTC | [35] |
|  |  | CR-P3 | ATTGGAGGGCAAGTCTGGTG | [35] |
|  |  | CPB-DIAGR | TAAGGTGCTGAAGGAGTAAGG | [35] |
